# Construction of Thirst Intervention Program for Awake Patients With Transoral Tracheal Intubation in ICU: A Mixed-Method Study

**DOI:** 10.1155/nrp/3635557

**Published:** 2025-10-22

**Authors:** Yingyang Li, Mengyuan Qiao, Linlin Hou, Xiumei Ding, Huanhuan Du, Enshe Jiang

**Affiliations:** ^1^Department of Critical Care Surgery, The First Affiliated Hospital of Henan University of Science and Technology, Luoyang 471003, China; ^2^School of Nursing, Henan University of Science and Technology, Luoyang 471023, China; ^3^Department of Critical Care Medicine, Henan Provincial People's Hospital, Zhengzhou University People's Hospital, Zhengzhou 450003, China; ^4^School of Nursing and Health, Henan University, Kaifeng 475004, China

**Keywords:** intensive care unit, intervention program, summary of evidence, thirst, tracheal intubation

## Abstract

**Background:**

Thirst is a common symptom of severe discomfort in awake patients with transoral tracheal intubation in the intensive care units (ICUs), which affects their physical and mental health. Due to the presence of transoral tracheal intubation, patients are unable to express their thirst needs in a timely manner through words, and nurses are prone to ignore their thirst symptoms. At present, there is a lack of systematic intervention protocols for managing thirst symptoms in awake patients undergoing transoral tracheal intubation in the ICU. Therefore, there is an urgent need to construct systematic intervention protocols for this group of patients.

**Design:**

A stepwise multimethod study was conducted. The intervention protocol was developed through qualitative research, evidence synthesis, and the Delphi method.

**Methods:**

Guided by symptom management theory, first, qualitative research methods were used to explore patients' thirst experience and nursing needs. Second, evidence-based methods were used to summarize the best evidence for thirst intervention in patients. Based on the results of qualitative research and evidence summary, a preliminary intervention protocol was formed. Finally, the Delphi method was used to consult 21 experts, soliciting their opinions and revising the protocol accordingly to form the final version.

**Results:**

The expert enthusiasm of the two rounds of expert consultation was 95.45% and 100%, respectively, the expert authority coefficient was 0.89, the Kendall harmony coefficient of item importance was 0.442 and 0.581, and the Kendall harmony coefficient of item feasibility was 0.363 and 0.509, respectively (*p* < 0.001). A final draft, including three aspects of thirst assessment, intervention strategies, and effectiveness evaluation, was developed.

**Conclusion:**

The thirst intervention program for awake patients with transoral tracheal intubation in ICU constructed in this study demonstrates sound scientific validity and can provide effective guidance for addressing patients' thirst symptoms while improving oral moisture and comfort levels.

## 1. Introduction

Transoral orotracheal intubation is a technique in which a specially designed endotracheal tube is inserted into the trachea through the mouth [1] and is the most commonly used method of establishing an artificial airway during resuscitation and mechanical ventilation of patients in intensive care units (ICUs). However, during the implementation process, it can cause the patient's mouth to be unable to close, increase the evaporation of water and saliva consumption in the patient's mouth, and make it difficult for the patient to communicate with medical staff in a timely manner using language [2]. This results in thirst symptoms being more common and overlooked in awake patients with transoral orotracheal intubation in the ICU than in other patients [3]. Research shows that 88.4% of patients with oral endotracheal intubation experience thirst during their wakefulness [4].

Thirst is a subjective feeling caused by physiological or behavioral factors that lead to a lack of water in the human body and cause a person's desire to drink, is an important part of the body's volume and fluid balance regulation mechanism, can be used to describe the intensity of thirst, frequency, duration and degree of pain [5, 6]. Studies have shown that thirst puts awake patients with transoral orotracheal intubation in the ICU in a state of intense stress such as pain, helplessness, and frustration, leading to tube biting and even unplanned extubation, increasing the patient's oxygen consumption and organ metabolic burdens, and affecting therapeutic efficacy [7]. Intense thirst lasting more than 24 h not only causes secondary oral mucosal infections and retro pulmonary infections in ICU patients awake via orotracheal intubation [8] but also increases their risk of delirium [9]. In addition, thirst can lead to post-traumatic stress disorder in awake patients undergoing transoral orotracheal intubation in the ICU, affecting their physical and mental health [10, 11]. However, thirst symptoms in awake patients with transoral orotracheal intubation in the ICU have not been adequately assessed and systematically intervened, and healthcare professionals pay less attention to their thirst symptoms [2].

Due to the presence of oral endotracheal intubation, patients are unable to express their thirst needs in a timely manner through language [2], and oral endotracheal intubation can lead to impaired swallowing function [12]. Therefore, methods such as drinking water orally, chewing gum, and taking ice cubes or popsicles to relieve thirst are not suitable for awake patients with transoral orotracheal intubation in the ICU. At present, in clinical practice, cotton swabs dipped in room temperature water are commonly used to moisten the patient's mouth, warm water is sprayed onto the patient's mouth, and there are currently studies exploring the method of ice water spraying onto the patient's mouth. However, these intervention measures are mainly unilateral and have varying effects, lacking comprehensive nursing measures. There are no unified intervention process and standards in the implementation process, and there is a lack of timely evaluation of patients' thirst status. A systematic thirst intervention plan has not yet been formed. Therefore, how to develop a systematic and feasible thirst intervention program for awake patients with transoral orotracheal intubation in the ICU has become an urgent clinical problem. The symptom management theory (SMT) was initially proposed in 1994 by the Symptom Management Group at the University of California, San Francisco School of Nursing, with subsequent updates in 2001 and 2008 [13]. Widely applied in managing various patient discomfort symptoms, SMT provides a valuable theoretical framework for alleviating or eliminating diverse symptoms [14]. The theory primarily comprises three dimensions: symptom experience, symptom management strategies, and symptom management outcomes. Therefore, guided by the SMT, this study developed a thirst intervention protocol for awake orotracheally intubated patients in the ICU, aiming to provide an evidence-based reference for addressing patients' thirst symptoms.

## 2. Methods

### 2.1. Study Design

**Figure 1:**
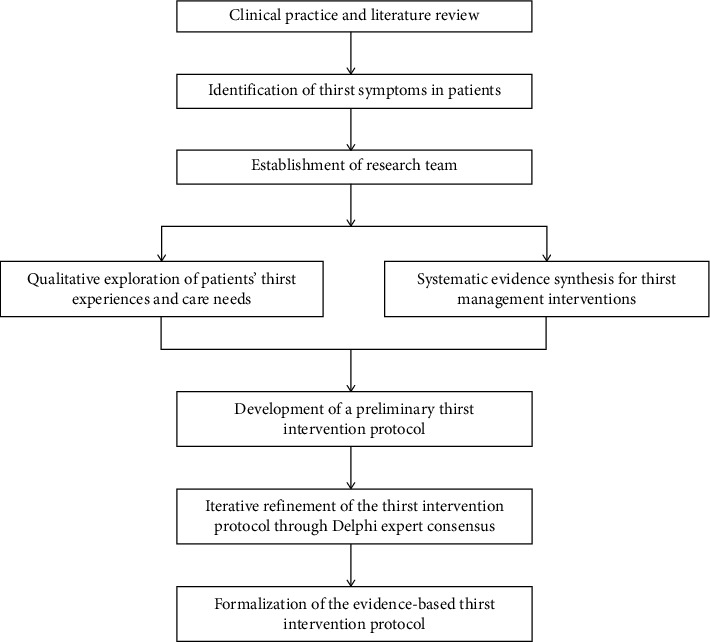
Study design flowchart.

The detailed study design flowchart is presented in Figure 1.

#### 2.1.1. A Research Team Was Formed

The research team comprised seven members: one nursing professor, one director of nursing department, one head nurse of ICU, one ICU physician, one ICU specialty nurse, and two nursing postgraduate students. The nursing professor and the director of the nursing department were responsible for overseeing the overall study design, quality control of the research, and selection of Delphi consultation experts. The ICU head nurse and physician were tasked with inviting Delphi consultation experts and revising the manuscript. One ICU specialty nurse and two nursing postgraduate students were responsible for conducting literature searches, performing qualitative interviews, collating and analyzing Delphi consultation results, and drafting and revising the manuscript. All research team members participated in the development of the Delphi expert consultation questionnaire and conducted the critical review of the manuscript content.

#### 2.1.2. Qualitative Research

This study employed purposive sampling to select patients who had undergone orotracheal intubation and were admitted to the ICU of a tertiary Grade A general hospital in Henan Province between September and October 2022. Semistructured interviews were conducted with these postextubation patients to comprehensively explore their thirst experiences and nursing care needs [15]. The interviews were conducted by two researchers. Prior to each interview, participants were fully informed of the study's purpose and significance, and their informed consent was obtained. All interviewees participated voluntarily in this study. Sampling continued until reaching thematic saturation, with 16 participants interviewed for 15–30 min each. The inclusion criteria for interviewees were as follows: age ≥ 18 years, conscious during orotracheal intubation with thirst experience, within 24–48 h post-extubation, Richmond Agitation–Sedation Scale (RASS) score of 0–1, and no history of auditory/visual impairments, with normal verbal communication ability. The exclusion criteria were as follows: history of oral cancer or oral surgery and presence of delirium. The interview guide covered the following: (1) Could you describe your thirst experience during orotracheal intubation in the ICU? (2) What specific difficulties did your thirst cause you? (3) Could you describe how you communicated your thirst symptoms to healthcare staff? (4) How effective were the healthcare providers' interventions in relieving your thirst? (5) Could you tell us what coping strategies you wished to use when experiencing thirst? (6) Is there anything else about your thirst experience that you think we should know? All interview data were transcribed and organized within 24 h postinterview, with dual independent verification. Two research team members subsequently analyzed the materials using Colaizzi's seven-step phenomenological analysis method. Based on the interview findings [15], the construction of the intervention protocol was informed by patients' expressed preferences, with detailed refinements made to the intervention content from the perspective of addressing patients' prioritized thirst care needs.

#### 2.1.3. Evidence Summary

Based on the 6S evidence resource pyramid model [16], top-down search of BMJ Best Practice, CINAHL, Cochrane Library, Clinical Trials, Embase, PubMed, UpToDate, Web of Science, Guidelines International Network (GIN), National Guideline Clearinghouse (NGC), National Institute for Health and Clinical Excellence (NICE) website, Australia's Joanna Briggs Institute (JBI) Centre for Evidence-Based Health Care database, Registered Nurses' Association of Ontario(RNAO) website, Scottish Intercollegiate Guidelines Network (SIGN), China National Knowledge Network, China Biomedical literature database, Wanfang database, Weipu database, Yimaitong, and other databases and websites' related evidence. The retrieval time limit is from the database establishment to November 20, 2022. When searching major guide websites or databases at home and abroad, the search term is “thirst/xerostomia/hyposalivation/asialia/mouth dryness/dry mouth.” When searching major websites and databases at home and abroad, the search terms were “intensive care units/intensive care unit^∗^/critical care/intensive care/critical illness/critical illnesses/critically ill,” “intubation, intratracheal/intratracheal intubation^∗^/endotracheal intubation^∗^/respiration, artificial/artificial respiration^∗^/mechanical ventilation^∗^,” “thirst/thirsts/xerostomia/xerostomias/hyposalivation^∗^/asialia^∗^/mouth dryness.” The inclusion criteria were as follows: (1) The target audience for the application of the evidence is awake patients with transoral orotracheal intubation in the ICU, (2) interventions are studies related to risk factor identification, assessment, and intervention for thirst, (3) the implementer of the evidence application is the healthcare professional, (4) the endpoints were patient thirst intensity, oral humidity, satisfaction, and complications, (5) evidence application site is ICU, and (6) the types of evidence are clinical decision-making, evidence summaries, guidelines, best practice, expert consensus, systematic evaluations, and randomized controlled trials. The exclusion criteria were as follows: (1) literature not in Chinese or English and (2) repeatedly published literature. Following rigorous literature screening and quality appraisal, 17 articles were ultimately included, yielding 26 best practice recommendations [17]. The preliminary intervention protocol was developed by integrating these evidence syntheses with qualitative findings, comprising three core components: symptom assessment, intervention strategies, and outcome evaluation.

#### 2.1.4. Expert Consultation

##### 2.1.4.1. Development of the Delphi Expert Consultation Questionnaire

The Delphi expert consultation questionnaire comprised three standardized sections: (1) Detailed questionnaire instructions were provided to panelists, encompassing the study's scientific rationale, objectives, clinical significance, and standardized completion guidelines. (2) The intervention protocol evaluation form incorporated dual assessment metrics for each item: importance and feasibility, rated on 5-point Likert scales (1 = “Not Important/Feasible” to 5 = “Extremely Important/Feasible”). Additionally, open-response fields were provided for expert recommendations regarding item modification, deletion, or supplementation. (3) Expert demographic characteristics: The survey captured panelists' professional profiles, self-rated familiarity with questionnaire content, and rationale for clinical judgments.

##### 2.1.4.2. Expert Panel Recruitment

The expert inclusion criteria were as follows: (1) bachelor's degree and above, (2) the professional title is intermediate and above, (3) 15 or more years of experience in the field of critical care nursing, (4) knowledge of transoral intubation and symptoms of thirst discomfort in critically ill patients, and (5) voluntary participation in this study. Based on the authority and accessibility of experts, 22 experts were selected. In the end, 21 experts completed the full process of expert consultation.

##### 2.1.4.3. Delphi Expert Consultation Implementation

The research team distributed and collected questionnaires via institutional email and WeChat platforms. Following the completion of Round 1 Delphi surveys, the research team systematically collated and analyzed response data. Through iterative discussions informed by predefined item selection criteria and expert qualitative feedback, modifications were implemented to generate the Round 2 questionnaire. Round 2 of the Delphi survey was conducted, during which the intervention protocol was iteratively refined based on expert panelists' quantitative ratings and qualitative feedback. Items were retained only if they met dual threshold criteria for both importance and feasibility: mean scores > 3.50 and coefficient of variation (CV) < 0.25 for each dimension.

### 2.2. Statistical Analysis

Data entry and statistical analyses were performed using SPSS 26.0. Continuous variables are presented as mean ± standard deviation, while categorical variables are summarized using frequencies and percentages. The expert positive coefficient is represented by the effective questionnaire recovery rate, where an effective recovery rate ≥ 70% indicates a satisfactory level of participation. The authority level of experts is represented by the authority coefficient (Cr), calculated as Cr = (Ca + Cs)/2, where Cr ≥ 0.7 is generally considered acceptable. The degree of expert coordination was assessed using the CV and Kendall's concordance coefficient (W). CV ≤ 0.25 indicated good consistency in expert opinions. The concentration of expert opinions was expressed by the mean ± standard deviation of importance and feasibility scores for each item, with higher scores indicating greater perceived importance or feasibility. Statistical significance was defined as *p* < 0.05.

### 2.3. Ethical Considerations

This study was conducted with the approval of the Ethics Committee of Henan University (Approval number: HUSOM2022-388) and in accordance with the principles of informed consent and confidentiality.

## 3. Results

### 3.1. Demographic Characteristics of Delphi Panel Experts

A total of 21 experts completed two rounds of the Delphi consultation, with a mean age of 49.43 ± 5.33 years and a mean working experience of 28.90 ± 7.06 years. The panel consisted of 16 full professors, four associate professors, and one intermediate-level expert.

### 3.2. Expert Engagement and Authority Level

In the first round, 22 electronic questionnaires were distributed with 21 valid responses returned, yielding an effective response rate of 95.45%. In the second round, all 21 distributed questionnaires were returned as valid responses, achieving a 100% effective response rate. The expert authority coefficient was 0.89.

### 3.3. Expert Consensus Level

In both rounds of expert consultation, all items achieved mean importance and feasibility scores > 3.5, with CV < 0.25. During the first round, Kendall's W values were 0.442 (importance) and 0.363 (feasibility) (both *p* < 0.001). The second round yielded improved concordance with *W* = 0.581 (importance) and *W* = 0.509 (feasibility) (both *p* < 0.001).

### 3.4. Results of the Expert Consultation

**Figure 2:**
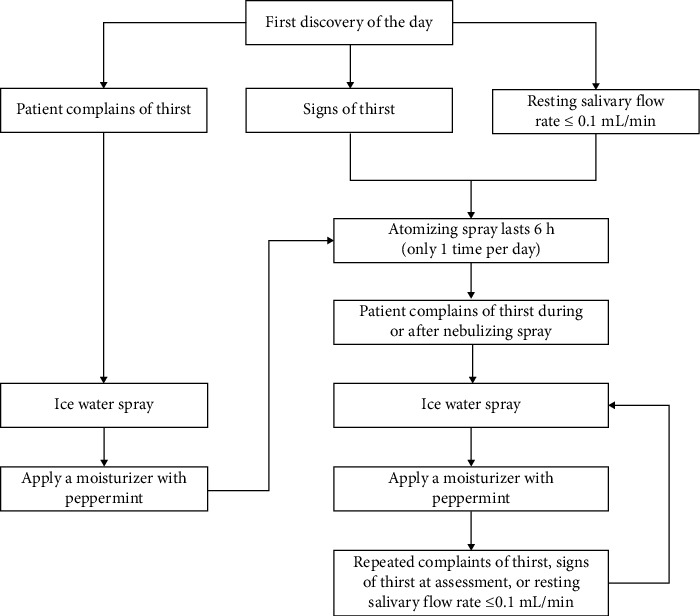
Intervention flowchart of the thirst intervention program for awake patients with transoral tracheal intubation in the ICU.

After the first round of expert consultation, according to the expert opinions, combined with the results of statistical analysis, and after discussion by the research group, the contents were deleted as follows: Delete the operable contents such as thick and greasy tongue coating, atrophy of tongue and nipple, and light of oral mucosa of palate in the entries on signs of thirst and delete the evaluation time of “patient's self-reported thirst,” which indicates that the patient has been thirsty, not signs of thirst. Modify the following: (1) Change symptom assessment to thirst assessment and symptom status to thirst symptom improvement and add hyperglycemia in laboratory tests. (2) The risk factors of thirst should also be assessed when the disease condition changes, the time to evaluate oral humidity should be increased, and the intensity of thirst should be assessed when the patient self-reports thirst. (3) Make clear the frequency of oral care, atomize the mouth when the humidity is poor, and give the specific value of the air bag pressure. After the second round of consultation, the following contents were revised: The content of no accumulation of saliva under the tongue was deleted from the signs of thirst, and oral care was performed at least once every 8 h. Finally, it was established that the thirst intervention scheme of awake patients in ICU after oral tube intubation was composed of three first-level items, 12 second-level items, and 24 third-level items. For details, please refer to Table 1 and Figure 2.

## 4. Discussion

### 4.1. The Developed Thirst Intervention Protocol for Awake Orotracheally Intubated ICU Patients Demonstrates Robust Scientific Validity and Clinical Reliability

This study, guided by SMT, employed qualitative research methods to investigate the thirst experience and nursing needs of awake orotracheally intubated ICU patients. The patient-centered findings provided a theoretical foundation for developing the intervention protocol, ensuring its clinical utility. This study further adopted an evidence-based approach to systematically synthesize the best available evidence for thirst intervention measures, thereby ensuring the scientific rigor of the developed protocol. Finally, the intervention protocol underwent rigorous validation through two rounds of Delphi expert consultation, ensuring its clinical applicability and methodological robustness. The expert panel was exclusively recruited from tertiary Grade A hospitals and academic medical centers affiliated with universities. The expert panel comprised 16 senior-title specialists and 14 postgraduate supervisors, with substantial clinical/academic experience (28.90 ± 7.06 years). The selected expert panel demonstrated strong representativeness and possessed in-depth domain-specific knowledge, with substantial theoretical expertise and clinical experience. Both rounds of the Delphi survey achieved questionnaire recovery rates exceeding 95%, demonstrating strong expert engagement and participation enthusiasm in this study. The expert authority coefficient (Cr) of 0.89 across both Delphi rounds confirms the reliability of the consultation results. The Kendall's concordance coefficients (W) for both importance and feasibility ratings of all items reached statistical significance (*p* < 0.001) across both Delphi rounds, while the CV remained below 0.25 throughout. These results demonstrate strong expert consensus and excellent coordination of panel opinions. In conclusion, this intervention protocol was developed through a rigorous methodological process, demonstrating robust scientific validity and clinical reliability for guiding evidence-based practice.

### 4.2. The Developed Thirst Intervention Protocol for Awake Orotracheally Intubated ICU Patients Provides Clinically Meaningful Guidance for Thirst Management

#### 4.2.1. Scheduled Assessment of Thirst Symptoms With Attentive Monitoring of Patients' Hydration Needs Is Recommended

The current research indicates that ICU nurses lack sufficient knowledge regarding thirst risk factors in awake orotracheally intubated patients, leading to suboptimal clinical recognition [2]. Therefore, this intervention protocol systematically identifies key risk factors for thirst and recommends standardized assessment during nursing shift handovers and upon clinical status changes, with mandatory documentation in handover records. Early and proactive identification of thirst risk factors coupled with targeted interventions can effectively prevent thirst symptoms, enhance oral comfort, and improve patients' willingness to cooperate with treatment [18]. Thirst, as a self-reported subjective symptom, can be promptly identified and effectively managed in patients with intact verbal communication abilities [19]. However, orotracheally intubated ICU patients face significant challenges in verbally expressing thirst symptoms and nursing needs, making it difficult for healthcare providers to promptly identify thirst, often resulting in considerable patient distress. This intervention protocol mandates systematic assessment of thirst indicators and oral humidity during all nursing shift handovers and prior to each oral care procedure, with standardized documentation integrated into handover records. Therefore, this intervention protocol provides clinical nurses with an evidence-based framework for timely identification of thirst-related distress and associated care needs.

#### 4.2.2. Development of Precise and Effective Thirst Intervention Strategies Significantly Alleviates Patients' Thirst Symptoms

Thirst-relief strategies can be categorized into preabsorptive and postabsorptive mechanisms [6, 20]. Preabsorption refers to the use of a small amount of low-temperature liquid to activate the cold receptor sensors on the trigeminal and glossopharyngeal nerves in the oral cavity before correcting plasma osmotic balance. This induces a pleasant cooling sensation in the body and transmits signals to the brain, creating a sense of drinking satisfaction. Postabsorption primarily involves replenishing the body with a large volume of fluid to maintain plasma osmotic balance, thereby alleviating thirst symptoms. For ICU patients with orotracheal intubation who cannot consume large amounts of water orally, preabsorption is a suitable method to alleviate their thirst symptoms. In this intervention protocol, the use of 0–6°C sterile ice water spray, 0.45% ice-cold sodium chloride solution via oxygen-driven nebulization, and menthol-based moisturizers all employ the preabsorption mechanism. These interventions are effective in relieving thirst symptoms in patients.

#### 4.2.3. Outcome Evaluation Should Be Implemented Throughout the Entire Thirst Intervention Process

Research indicates that some orally intubated ICU patients report the thirst-relief interventions provided by nurses to be ineffective, with persistent thirst even after receiving antithirst measures [3]. Therefore, after implementing thirst-relief interventions for orally intubated ICU patients, healthcare providers should proactively obtain patient feedback, promptly evaluate the effectiveness of the interventions, and continuously refine thirst alleviation measures. If the interventions prove ineffective and lack timely outcome evaluation, they will fail to alleviate patients' thirst symptoms. This may even lead to patients developing distrust toward healthcare providers, resulting in strained clinician–patient relationships that ultimately compromise patients' compliance with treatment regimens [21]. Therefore, outcome evaluation should be integrated throughout the entire process of thirst intervention for patients. This intervention protocol evaluates the effectiveness of thirst-relief measures primarily through the following parameters: thirst intensity, oral moisture, oral comfort, and complication rates. In clinical practice, nurses should assess the outcomes of thirst alleviation interventions based on these key indicators.

## 5. The Following Work of the Research

Future multicenter studies with larger sample sizes are warranted to validate the intervention protocol's efficacy and facilitate its iterative refinement. The current thirst assessment for conscious orotracheally intubated ICU patients lacks standardized tools and relies predominantly on subjective measures. Future research should prioritize developing integrated assessment instruments that combine subjective symptom reporting with objective biomarkers to enable precise thirst identification. Regarding thirst intervention, while the current protocol incorporates ice water spray as an evidence-based measure, its administration frequency is not yet stratified according to thirst intensity levels. Future studies should establish optimal intervention frequencies tailored to graded thirst severity to achieve precision symptom relief. Regarding patient communication, the presence of orotracheal intubation creates significant verbal communication barriers, impairing timely expression of thirst-related needs. Future research should focus on developing validated communication strategies tailored to this vulnerable population.

## 6. Theoretical and Methodological Contributions

The current research on thirst in awake, orally intubated ICU patients primarily focuses on prevalence surveys and influencing factor analyses. Despite the existing interventions, there remains a critical gap in (1) comprehensive symptom assessment methodologies, (2) standardized multimodal intervention protocols (with reported efficacy varying significantly across studies), and (3) evidence-based precision care frameworks. To our knowledge, no systematic thirst management program has been established for this vulnerable population. This study systematically developed an evidence-based thirst intervention protocol for conscious orotracheally intubated ICU patients, establishing a standardized framework for comprehensive symptom assessment and structured intervention delivery, which not only addresses this prevalent yet undermanaged clinical issue but also contributes theoretically to advancing precision nursing practice in critical care settings through its measurable outcomes and implementable design.

Methodologically, this study employed a tripartite approach to develop the intervention protocol: (1) initial qualitative exploration of thirst experiences and care needs among conscious orotracheally intubated ICU patients, ensuring patient-centered practicality, (2) systematic evidence synthesis to establish recommendations for thirst management, guaranteeing scientific rigor, and (3) iterative Delphi expert consultation for protocol refinement, confirming clinical feasibility. The innovative integration of phenomenological inquiry, evidence-based methodology, and expert consensus procedures represents a methodological advancement in nursing intervention development.

## 7. Implications for Policy and Practice

Thirst is a common discomfort among conscious ICU patients with orotracheal intubation, severely impacting their physical and mental health. However, healthcare providers currently pay insufficient attention to thirst symptoms, failing to conduct regular assessments and systematic interventions in clinical practice. This intervention protocol is developed based on the thirst experiences of conscious orotracheally intubated ICU patients, existing evidence on thirst interventions, and expert recommendations, incorporating insights from patient needs, current evidence, and expert consensus. From a policy perspective, this study advocates for higher-quality nursing care, ensuring adequate patient-centered compassion, prioritizing thirst management, and incorporating thirst assessment into routine shift handovers. It also calls for enhanced training on thirst-related knowledge for medical staff. In clinical practice, the study recommends regularly assessing patients' thirst-related care needs, implementing timely thirst interventions, and proactively addressing thirst to improve patient comfort.

## 8. Limitations

Because of research funding constraints and consideration of patients financial burden, the less expensive resting salivary flow rate method was used to assess patients oral humidity in this intervention protocol, and the simpler but more expensive oral humidity tester was not used, making the process of assessing oral humidity relatively cumbersome. Future researchers should conduct large-scale, multicenter studies to further validate the efficacy of this intervention protocol, with the goal of continuous refinement and optimization.

## 9. Conclusion

Guided by the SMT, this study developed an evidence-based thirst intervention protocol for conscious orotracheally intubated ICU patients, integrating findings from qualitative research on thirst experience with a systematic synthesis of existing clinical evidence. This protocol was rigorously developed through a standardized Delphi consensus process, demonstrating both scientific validity and clinical applicability for guiding evidence-based nursing practice in this specialized patient population.

## Figures and Tables

**Table 1:** Thirst intervention program for awake patients on transoral intubation in ICU.

First-level entry	Second-level entry	Third-level entry
1. Thirst assessment	1.1. Risk factors for thirst	1.1.1. Content of the assessment:
		Disease factors: The more severe the patient's condition, the more likely it is to induce symptoms of thirst, especially symptoms such as vomiting, diarrhea, bleeding, high fever, and diseases such as heart failure, renal failure, and diabetes
		Laboratory tests: low calcium, high sodium, high blood sugar, elevated hemoglobin levels
		Pharmacologic factors: diuretics, e.g., furosemide > 60 mg, opioids > 50 mg, nonsteroidal anti-inflammatory drugs, corticosteroids, antihypertensive drugs, anticholinergics, proton pump inhibitors, 5-hydroxytryptamine reuptake inhibitors, neuroleptics, tricyclic antidepressants
		Therapeutic factors: negative fluid balance
		1.1.2. Timing of assessment: Assessments are made once per shift during the nurse's handover and when the patient's condition changes and are included in the handover
	1.2. Signs of thirst	1.2.1. Assessment: peeling or chapped lips, bleeding from broken oral mucosa
		1.2.2. Timing of the assessment: one assessment per shift during the nurse handover and before each oral care session and included in the handover
	1.3. Oral humidity	1.3.1. Evaluation method: evaluate the oral humidity by measuring the resting saliva flow rate in the patient's mouth, that is, first use a dry cotton swab to dry the saliva in the patient's mouth, then take three preweighed dry cotton swabs and place them on the patient's bilateral parotid glands and under the tongue, take out the cotton swab and weigh it 2 min later, calculate the difference between the two weights of the cotton swab before and after to obtain the resting saliva flow rate, and convert its unit into mL/min (1 g = 1 mL). When the resting saliva flow rate is ≤ 0.1 mL/min, it indicates that the patient's mouth is dry. The lower the resting saliva flow rate is, the drier the patient's mouth is, and the more likely to have thirsty symptoms. After evaluation, give the patient ice water spray one intervention
		1.3.2. Timing of assessment: one assessment per shift after the nurse handover and before each oral care session and included in the handover
	1.4. Thirst intensity	1.4.1. Assessment tools and methods: the patients' thirst intensity is assessed with the numerical thirst assessment scale, which adopts a 0–10 point system to indicate the patients' thirst intensity, and the patients can choose one score according to their own thirst intensity, with 0 being No Thirst, 1–3 being Mild Thirst, 4–7 being Moderate Thirst, and 8–10 being Severe Thirst. Due to the verbal communication barriers in awake patients with transoral intubation in the ICU, when using this assessment tool, patients with normal muscle strength can use a pen to select their own corresponding thirst intensity score, and nurses can help patients who cannot hold a pen to point out their corresponding thirst intensity score and have them nod their heads to confirm
		1.4.2. Timing of assessment: patients were assessed when they complained of thirst. Patient self-complaints of thirst included, but were not limited to, the following: thirst gestures, open-mouth signaling, nodding after rhetorical questions, and writing

2. Intervention strategies	2.1. Oral care	2.1.1. Content of the intervention: patients were given oral care according to clinical norms during transtracheal intubation
		2.1.2. Frequency of intervention: oral care at least every 8 h
	2.2. Ice water spray	2.2.1. Intervention method: spray the patient's oral cavity with ice sterilized water for injection spray at 0–6°C, the sprayed parts are the palate, left cheek, tongue, right cheek, and throat, and each part is sprayed three times in a row, and the amount of liquid sprayed each time is about 0.1 mL, and the total amount of liquid in the first intervention is about 1.5 mL. Before the intervention, the patient should be reminded again that the temperature of the sterilized water for injection is “0–6°C” so that the patient can be psychologically prepared for the icy water spraying in advance, and to avoid stimulation of the patient by the low temperature of the water
		2.2.2. Timing of intervention: one ice water spray was performed when the patient showed “signs of thirst or resting salivary flow rate ≤ 0.1 mL/min or complained of thirst,” and the ways in which the patient complained of thirst included, but were not limited to, the following: thirst gesture, open-mouth sign, nodding after asking a question and writing, etc. The position of the transtracheal intubation was determined before and after the intervention, and the pressure of the airbag was ensured to be within the range of 25–30 cm H_2_O
	2.3. Atomizing spray	2.3.1. Intervention method: 0.45% sodium chloride solution is configured according to the ratio of saline 5 mL + sterilized water for injection 5 mL, with the dental pads placed at the patient's mouth and lips as the center, the nebulizer is placed at a position 5 cm away from the patient's dental pads and aimed at the patient's mouth and lips, and the nebulizer is driven by the atomized spray with the 0.45% iced sodium chloride solution at 6–10°C, with 6–8 L/min oxygen drive for atomization spray, in order to maintain the patient's oral cavity and its surrounding environment of the degree of wetness, nebulization during the close attention to the patient's response and tubing fixed situation
		2.3.2. Timing of intervention: nebulization spraying was started when the patients showed signs of thirst or had a resting salivary flow rate of ≤ 0.1 mL/min or complained of thirst for the first time every day. Before and after the intervention, the position of transoral intubation was determined and the pressure of the airbag was ensured to be within the range of 25–30 cm H_2_O
		2.3.3. Duration of intervention: continuous atomized spraying for 6 h (which can be carried out automatically after the start of the atomized spraying, during which it is only necessary to replace the liquid in the nebulizer), 1 time per day
	2.4. Apply a moisturizer containing peppermint	2.4.1. Intervention method: apply a moisturizer containing peppermint to the patient's lips and mouth; other types of moisturizers may be used for those who are allergic or intolerant to peppermint
		2.4.2. Timing of the intervention: at the end of each oral care session and after the ice water spray intervention
	2.5. Psychological care	2.5.1. Intervention content: implement humanistic care and targeted psychological care, establish a good nurse–patient relationship with the patients, explain to the patients the reasons why they are often thirsty and cannot drink normal water through their mouths, and eliminate their anxiety, fear, stress, and other psychological emotions
		2.5.2. Timing of intervention: psychological care was provided when patients complained of thirst, and the ways in which patients complained of thirst included, but were not limited to, the following: thirst gestures, open-mouth signaling, nodding after rhetorical questions, and writing

3. Evaluation of effectiveness	3.1. Improvement of thirst symptoms	3.1.1. Intensity of thirst: evaluated after the intervention using the numerical thirst rating scale
		3.1.2. Oral moisture: evaluated after the intervention by measuring the resting salivary flow rate in the patient's mouth
	3.2. Oral comfort	3.2.1. Oral comfort: after the intervention, oral comfort was evaluated with the oral comfort numerical rating scale, which used a 0–10 point system to indicate the patient's oral comfort, with 0 being Very Uncomfortable and 10 being Very Comfortable, and the larger the score, the higher the patient's oral comfort. Due to the ICU transoral intubation awake patients with verbal communication barriers, in the use of this assessment tool, patients with normal muscle strength can use a pen to select their own corresponding oral comfort scores, and patients who cannot hold a pen, the nurse can help them to point out the corresponding oral comfort scores so that they can nod to confirm
	3.3. Complications	3.3.1. Incidence rate of unplanned extubation for tracheal intubation: (number of unplanned extubation for tracheal intubation occurring in the same patients after the intervention/total number of tracheal intubation placements in the same patients after the intervention) × 100%, which was evaluated by this formula after the intervention
		3.3.2. Incidence of choking cough: (number of people experiencing choking cough during the intervention period/total number of people in the intervention) × 100%, which was evaluated after the intervention using this formula. Choking cough is the irritating cough that occurs in patients during the intervention

## Data Availability

The data are not publicly available due to privacy or ethical restrictions.
